# Molecular mechanisms of semaglutide and liraglutide as a therapeutic option for obesity

**DOI:** 10.3389/fnut.2024.1398059

**Published:** 2024-04-29

**Authors:** Rafael Tamayo-Trujillo, Viviana A. Ruiz-Pozo, Santiago Cadena-Ullauri, Patricia Guevara-Ramírez, Elius Paz-Cruz, Raynier Zambrano-Villacres, Daniel Simancas-Racines, Ana Karina Zambrano

**Affiliations:** ^1^Centro de Investigación Genética y Genómica, Facultad de Ciencias de la Salud Eugenio Espejo, Universidad UTE, Quito, Ecuador; ^2^Universidad Espíritu Santo, Samborondón, Ecuador; ^3^Centro de Investigación de Salud Pública y Epidemiología Clínica (CISPEC), Universidad UTE, Quito, Ecuador

**Keywords:** obesity, molecular mechanism, semaglutide, liraglutide, weight loss

## Abstract

Obesity, a chronic global health problem, is associated with an increase in various comorbidities, such as cardiovascular disease, type 2 diabetes mellitus, hypertension, and certain types of cancer. The increasing global prevalence of obesity requires research into new therapeutic strategies. Glucagon-like peptide-1 receptor agonists, specifically semaglutide and liraglutide, designed for type 2 diabetes mellitus treatment, have been explored as drugs for the treatment of obesity. This minireview describes the molecular mechanisms of semaglutide and liraglutide in different metabolic pathways, and its mechanism of action in processes such as appetite regulation, insulin secretion, glucose homeostasis, energy expenditure, and lipid metabolism. Finally, several clinical trial outcomes are described to show the safety and efficacy of these drugs in obesity management.

## Introduction

Obesity is a chronic disease, and its prevalence has increased worldwide (1). According to the World Health Organization (WHO), there were approximately 1.9 billion overweight adults in 2016, of which 650 million were obese (2, 3). Genetic, environmental, and behavioral interactions have been linked to obesity; thus, the consumption of high-calorie foods associated with sedentary lifestyles increases the number of obese people in the world (4). Therefore, obesity can lead to a high risk of cardiovascular diseases, type 2 diabetes mellitus (DM2), hypertension, and some types of cancer. In addition, other diseases, such as dyslipidemia and even depression, are associated with obesity (5, 6).

Despite the increase in weight management approaches, the pandemic of obesity is still rising. However, strategies to control this phenomenon have primarily focused on physical exercise, behavioral factors, surgery (including bariatric surgery), and pharmaceutical interventions (7).

**Figure 1:**
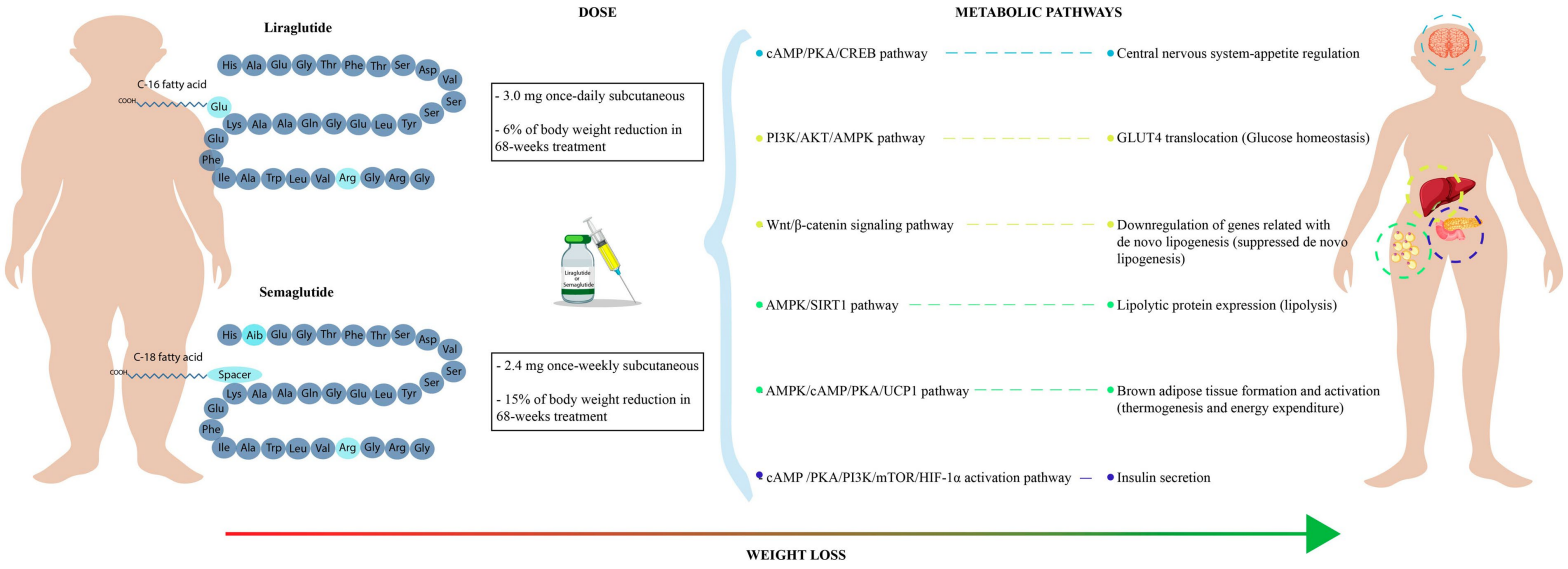
Metabolic outcomes of semaglutide and liraglutide treatment. It shows the molecular structure and recommended dose of GLP-1RA (semaglutide and liraglutide). Mechanisms of action of semaglutide and liraglutide in several metabolic pathways related with body weight loss.

Pharmacological strategies have been fundamental for reducing the obesity pandemic (8). In this context, the group of glucagon-like peptide 1 receptor agonists (GLP-1RA), which mimic the natural hormone glucagon-like peptide 1 (GLP-1), has been mainly used for the treatment of DM2 (9). In addition, studies have shown that these drugs can have pleiotropic effects such as blood pressure lowering, weight reduction, endothelial protection, and insulin sensitivity (Figure 1). For instance, semaglutide and liraglutide, two GLP-1RA agonist drugs, have been associated with weight loss and body weight maintenance (10–12).

The present review aims to elucidate and describe the molecular mechanisms of the GLP-1RA drugs, semaglutide and liraglutide, and their effectiveness and potential as a therapeutic option against obesity.

## Methodology

The scientific papers search was performed in Google Scholar and PubMed databases. The following individual and combined search terms were used: “GLP-1,” “Semaglutide and Liraglutide,” “molecular mechanism,” “lipid metabolism,” “insulin metabolism,” “glucose homeostasis,” “energy expenditure” “obesity treatment,” “type 2 diabetes mellitus,” “side effects,” “clinical trials,” “treatment option,” “fatty liver disease,” “hypertension,” “neurodegenerative diseases,” “cardiovascular diseases,” “therapeutic options.” Only articles published in the last 10 years are included, although older pivotal studies were added.

## Molecular mechanisms of semaglutide and liraglutide

### GLP-1 receptor activation

GLP-1 is an incretin hormone secreted by enteroendocrine L-cells and α-cells in the pancreas and central nervous system. GLP-1 activates insulin secretion in response to elevated plasma glucose levels (13). In addition, GLP-1 activation increases neogenesis and proliferation while decreasing apoptosis of pancreatic β-cells (14). This GLP-1 hormone binds to the G protein-coupled GLP-1 receptor (GLP-1R) and activates key intracellular metabolic pathways. For instance, the GLP-1 hormone can trigger the adenylate cyclase (AC) metabolic pathway, leading to elevated levels of intracellular cyclic AMP (cAMP), subsequently activating the protein kinase A (PKA). PKA promotes exocytosis of insulin-containing vesicles from pancreatic β-cells and increases glucose-dependent insulin secretion (15). Conversely, GLP-1 can inhibit glucagon release from pancreatic α-cells, decreasing liver production of glucose (16).

Furthermore, cAMP-dependent mechanisms acting through PKA, and the exchange protein directly activated by cAMP (EPAC) inhibit ATP-dependent potassium channels and increase the activity of calcium channels. The influx of more calcium ions into the cell activates glucose-induced membrane depolarization and elevated cell sensitivity to glucose (17).

In addition, GLP-1 can stimulate the epidermal growth factor (EGF), triggering the phosphatidylinositol-3 kinase (PI3K), which activates transcription factors associated with β-cell growth while inhibiting those linked to β-cell apoptosis (18).

Moreover, these mechanisms are a consequence of Gβγ signaling, facilitated by the interaction of GLP-1R with distinct G proteins, which can activate various metabolic pathways using the identical ligand. Consequently, the metabolic pathways initiated by Gα proteins within GLP-1R involve cAMP activation, extracellular signal-regulated kinases 1 and 2 (ERK 1/2) and increased intracellular calcium levels (17, 19).

### Chemical structure of semaglutide and liraglutide and its interaction with GLP-1 receptor

One of the pharmacological approaches against diseases like diabetes and obesity involves prolonging the time of action of GLP-1. For example, some GLP-1RA drugs, such as semaglutide and liraglutide, have already been approved by the United States Food and Drug Administration (FDA) for DM2 and chronic weight control (20).

Semaglutide and liraglutide were derived from the native structure of GLP-1. Semaglutide is a drug with 94% homology with human GLP-1. Its chemical structure consists of 31 amino acids with two amino acid substitutions (Aib8 and Arg34). Notably, the substitution at position 8 reduces susceptibility to degradation by dipeptidyl peptidase-4 (DPP-4) (21, 22). Conversely, liraglutide, another GLP-1RA, has 97% homology with human GLP-1. Its chemical structure, unlike semaglutide, has an Arginine-Lysine substitution at position 34 and a fatty acid residue is attached to Lysine 26. This modification enhances albumin binding, contributing to an increase in the half-life of the drug to 11 to 13 h (14).

Both, semaglutide and liraglutide, by binding to GLP-1R, can activate cAMP and PKA signaling cascades. As a result, there is an increased insulin secretion in the pancreatic β-cells and suppression of glucagon release. Furthermore, these pathways have also been linked to appetite regulation, modulation of gastric emptying, and cardiovascular effects (23, 24).

### Semaglutide and liraglutide effects on central nervous system regulation

GLP-1 and GLP-1RA are involved in the activation of various central nervous system (CNS) processes, including satiety, thermogenesis, blood pressure, neurogenesis, and inflammation reduction (15). GLP-1 increases the expression of the cAMP, PKA, and CREB metabolic pathways. Additionally, GLP-1 modulates the phosphorylation of AKT, ERK, GSK-3B, and mTOR to increase cell viability and growth (25, 26).

Obesity has been associated with chronic inflammation, and GLP-1RA can modulate these inflammatory processes (27). GLP-1R is present in macrophages, lymphocytes, and monocytes which regulate immune cell signaling by suppressing proinflammatory cytokines such as TNF-α, IL-6, and IL-1β (28). In addition, studies have shown that GLP-1RAs decrease oxidative stress and improve endothelial function contributing to a reduction of inflammation in obesity (29).

Moreover, GLP-1 promotes neurogenesis by reducing inflammation and decreasing the expression of proinflammatory cytokines IL-6, IL-10, and microglial activation (25). GLP-1R is present in different brain regions, such as the cerebral cortex, thalamus, hypothalamus, substantia nigra, and cerebellum, having crossed the blood–brain barrier, influencing processes like appetite control and satiety (15). The GLP-1RA drugs semaglutide and liraglutide, due to their longer half-lives, may have more prolonged effects in the CNS and regulate appetite metabolic processes (30).

Studies have observed that GLP-1RA decreases food intake, slows gastric emptying, and promotes the release of hormones such as leptin and peptide YY, involved in satiety (7). The hormone leptin is fundamental in the regulation of body weight since it suppresses the orexigenic (appetite-inducing) pathway and activates the anorexigenic (satiety-inducing) pathway (8). Therefore, GLP-1RA may target different pathways to control appetite and inflammatory processes which would contribute to the treatment of obesity.

### Metabolic effects

#### Semaglutide and liraglutide on insulin secretion

The effect of GLP-1 and GLP-1RA in pancreatic β-cells promotes glucose catabolism and insulin secretion (15, 31). This process relies on the mTOR-dependent HIF-1α activation pathway, which starts with the binding of the GLP-1 to the GLP-1R, activating the AC. Subsequently, AC increases the cAMP expression, which promotes the activation of both, PKA and EPAC. Activated PKA promotes the PI3K/mTOR pathway in β-cells, leading to the activation of the Hypoxia-Inducible Factor 1 (HIF-1). The transcriptional factor HIF-1 induces glycolytic genes activation in response to hypoxia and growth factors. Enhanced glycolysis facilitates citric acid cycle activation, elevating intracellular ATP concentration. High ATP amounts induce the closure of potassium channels, depolarizing the cell membrane, leading to calcium influx and insulin vesicle release. This release can be rapid, with a peak at 10 min post-initial glucose stimulus, followed by a sustained release from major insulin granules (approximately 90–95%) lasting up to 60 min under normal conditions (15, 31). However, the low half-life of GLP-1 incretin could affect this process in individuals with DM2 or obesity (32, 33). Therefore, GLP-1RA drugs like liraglutide or semaglutide can counteract DPP-4 degradation, prolonging the GLP-1R signal and maintaining insulin secretion via the mTOR-dependent HIF-1α pathway (34).

#### Semaglutide, liraglutide and glucose homeostasis

Liraglutide and semaglutide are mainly prescribed for the treatment of DM2 (34). These GLP-1RA drugs can improve blood glucose levels and reduce body weight (14, 35). There are various mechanisms involved in glucose homeostasis, like glucose-dependent insulin secretion, insulin biosynthesis, and glucagon regulation (34). Moreover, studies have shown that GLP-1RA could regulate glucose homeostasis in an insulin-independent mechanism via the 5′-AMP-activated protein kinase (AMPK) pathway (36, 37). The AMPK pathway is activated by the PI3K/AKT pathway, which promotes the translocation of the glucose transporter 4 (GLUT4) from intracellular vesicles to the plasma membrane. The increased levels of GLUT4 in the plasma membrane stimulate glucose uptake, promoting glucose homeostasis (36–38).

### Lipid metabolism

#### Semaglutide and Liraglutide effects on lipid metabolism

GLP-1RA drugs have been associated with reduced food intake and body weight loss (39). The molecular mechanisms involve the stimulation of the Wnt/β-catenin signaling pathway by GLP-1R. Activation of the Wnt/β-catenin pathway has negative effects on adipogenesis by downregulating the expression of genes related to *de novo* lipogenesis. These downregulated genes include *DGAT1, SCD1, ApoB, FABP1,* and *FOXA1*, which are involved in fatty acid and triglycerides synthesis. Moreover, a decreased expression of the *FABP1* and *FOXA1* genes leads to decreased uptake of free fatty acids. The AMPK pathway also plays a role in inhibiting lipogenesis by regulating lipogenic genes like acetyl-CoA carboxylase. Inhibition of the acetyl-CoA carboxylase suppresses the *de novo* lipogenesis and improves fatty acids oxidation (37, 40).

The effect of GLP-1RA drugs in adipose tissue involves the lipolytic process. These agonists stimulate the AMPK pathway, leading to the activation of Sirtuin 1 (SIRT1), a NAD^+^-dependent deacetylase that upregulates the expression of lipolytic proteins like triacylglycerol lipase. Consequently, triglyceride depletion occurs in white adipose tissue, resulting in reduced fat accumulation and improved energy expenditure. Moreover, GLP-1RA negatively influences the expression of peroxisome proliferator-activated receptors (PPARs), leading to a downregulation of proteins associated with lipid metabolism (37, 41, 42).

#### Semaglutide and liraglutide in thermogenesis and energy expenditure

GLP-1 and GLP-1RA stimulate the GLP-1R in the central nervous system to modulate lipid metabolism in white and brown adipose tissues leading to a reduction in body weight. This process involves AMPK activation in the hypothalamic ventromedial nucleus by GLP-1 or GLP-1RA, promoting brown adipose tissue thermogenesis and white adipose tissue browning. The activated AMPK pathway enhances brown tissue activation via transcriptional regulators of genes involved in brown tissue development (PR domain containing 16, peroxisome proliferator-activated receptor γ, peroxisome proliferator-activated receptor γ coactivator 1α). Moreover, AMPK stimulates the activation of the cAMP/PKA pathway in brown adipocytes, triggering lipolysis through the release of free fatty acids and upregulation of uncoupling protein 1 (UCP1). Additionally, the cGMP second messenger is involved in mitochondrial biogenesis and UCP1 activation in brown adipose via the nitric oxide-sensitive soluble guanylyl cyclase pathway. UCP1 is essential for releasing electrons during oxidative phosphorylation in the inner mitochondrial membrane, generating heat (thermogenesis). Increased expression of UPC1 and a higher number of mitochondria are characteristics of activated brown adipose tissue, facilitating the lipolytic cycle (43–45).

Another effect of GLP-1RA is the increase of sympathetic nervous system (SNS) activity. Activated SNS promotes brown adipose tissue thermogenesis and white adipose tissue browning via hypothalamic AMPK activity. Furthermore, the thyroid hormone (TH) is an essential regulator of brown adipose tissue activity. In this context, GLP-1RA drugs could activate the thermogenic proteins in brown adipocytes, increasing intracellular type 2 deiodinase (D2) expression and subsequent TH activation. Therefore, TH activates brown adipocytes and promotes oxygen consumption and thermogenesis (43, 46, 47).

## Discussion

The metabolic effect of semaglutide and liraglutide in obese people is remarkable. The main mechanism of action of these GLP-1RA is the stimulation of the GLP-1R that triggers the activation of several metabolic pathways involved in insulin secretion, lipid metabolism, energy expenditure, pro-survival and anti-apoptotic cellular signaling, and oxidative stress prevention, in several tissues like the pancreas, central nervous system, heart, muscle, kidneys, gut, among others (15). The wide distribution of the GLP-1R has allowed the study of semaglutide and liraglutide pharmacological protocols to improve metabolic diseases in several experimental models (26, 40, 48–50). The assessment of these GLP-1RA could elucidate the role of these drugs in the cellular signaling process of obese-related diseases, which could support the development of new pharmacological approaches. For instance, the combination of GLP-1RA and enzyme inhibitors of proinflammatory, lipogenesis, pro-apoptotic, and pro-oxidative stress processes could be evaluated in metabolic diseases. Although, rigorous safety and efficacy-controlled trials must be carried out, like those performed to assess the effect of semaglutide and liraglutide in obese or DM2 individuals.

Clinical trials have evaluated the effectiveness of GLP-1RA drugs, semaglutide, and liraglutide, in treating obesity. The Semaglutide Treatment Effect in People with Obesity (STEP) program evaluated the once-weekly administration of 2.4 mg subcutaneous semaglutide in people with obesity or overweight. In the STEP 1 trial, 1961 participants underwent dietary and exercise interventions. In this trial, 69 to 79% of the participants lost weight with an average weight reduction of ≥10% after 68 weeks of treatment compared with placebo group (12–17%) (51). Additionally, the STEP trials evaluated patients with obesity and DM2; the results supported the recommended dose of 2.4 mg of semaglutide per week for weight loss in individuals with obesity, with or without DM2 (52).

Similarly, in the Peptide Innovation for Early Diabetes Treatment (PIONEER) clinical trial, oral semaglutide was tested in patients with DM2. Results showed that oral semaglutide has a significantly higher efficacy compared to placebo and other DPP4 inhibitor drugs (53). Moreover, the results from the PIONEER clinical trials suggested that oral semaglutide effectively reduced HbA1c and body weight, showing responsiveness in diverse age groups with a diagnosis of DM2 (54).

The efficiency of liraglutide was evaluated in the clinical trial called SCALE (Satiety and Clinical Adiposity: Evidence for liraglutide), where it was determined that a dosage of 3.0 mg induced an 8% weight loss equivalent to 8.4 kg of the initial weight in over 56 weeks. In comparison, the placebo group experienced a decrease of 2.6% equivalent to 2.8 kg. Participants also improved weight-related comorbidities through lifestyle and diet adjustments (55).

Azuri et al. showed that semaglutide had a better performance than liraglutide in terms of weight loss (12.4% [95% CI: 11.5–13.4%] vs. 5.4% [95% CI: 5–5.8%]). Moreover, this study reported that semaglutide had a lower economic spend than liraglutide for obesity management (56). This highlights the potential use of semaglutide due to its low cost and superior performance.

The posology of semaglutide and liraglutide also showed differences in body weight reduction. In obese individuals without diabetes mellitus, a once-weekly subcutaneous semaglutide (2.4 mg) dose had a significant body weight change compared with a once-daily subcutaneous liraglutide (3.0 mg) dose after 68 weeks. Participants under the semaglutide treatment showed a 15% weight loss than baseline, while liraglutide recipients experienced a 6% weight loss (57). Therefore, semaglutide also has an advantage due to the smaller number of doses and greater weight loss.

A meta-analysis revealed that a 2.4 mg dose of semaglutide led to a reduction of 12.4 kg of body weight, while doses of 3.0 mg of liraglutide, 1.0 mg of semaglutide, and 1.8 mg of liraglutide led to reductions in body weight of 5.2 kg, 3.7 kg, and 1.8 kg, respectively. The evaluation of these doses was performed in various periods, ranging from 20 weeks to 68 weeks. The most effective treatment involved a weekly dose of 2.4 mg semaglutide for 68 weeks. Moreover, for the dose of 2.4 mg semaglutide, the decrease in glycated hemoglobin was the highest (1.48% reduction with 2.4 mg semaglutide dose) compared with the other doses (58). Once again, semaglutide demonstrates a higher performance compared with liraglutide, although individual medical status must be assessed before the prescription of this treatment to prevent side effects.

Another important component of the treatment with GLP-1RA is the inclusion of lifestyle interventions, including exercise and a low-calorie diet (1, 59). This factor is significant for weight loss sustaining, given that after subcutaneous semaglutide (2.4 mg) treatment suspension, studies have shown that there may be a weight regain worsening cardiometabolic parameters (60). This highlights that GLP-1RA treatment must be continuous, along with a healthy lifestyle. Therefore, monitoring after several years of semaglutide or liraglutide withdrawal should be performed to verify metabolic status and weight. For instance, the outcomes of semaglutide and liraglutide in patients with DM2 have shown a cardiovascular risk factors reduction and an improvement in glucose levels and nutritional status (22, 58, 61–63).

The safety profile of GLP-1RA drugs generally indicates a low incidence of side effects, although it depends on the dose and the administration mode. For instance, oral semaglutide (14 mg) exhibited more side effects (such as vomiting or gastrointestinal issues) than subcutaneous liraglutide (1.2 mg); however, these side effects were no different from those observed with subcutaneous semaglutide treatment (64). Moreover, the use of semaglutide has been related to the increased risk of cholelithiasis (65).

The effectiveness of these GLP-1RA drugs, especially semaglutide, is associated with an improvement in other diseases like cardiovascular diseases (22, 50). Studies suggest that DM2 accompanied by insulin resistance may contribute to memory impairment and the development of neurodegenerative diseases such as Alzheimer’s and Parkinson’s disease (66). Furthermore, these GLP-1RA medications have also been related to a reduction of neuroinflammation, probably due to its possible role in neuronal insulin signaling pathway restoration (49, 67, 68). Hence, semaglutide and liraglutide could also have the potential to ameliorate neurodegeneration processes observed in pathologies like Alzheimer’s and Parkinson’s diseases, although further research is needed (48, 69). Moreover, clinical trials have reported a reduction of the inflammatory C-reactive protein (70), which could indicate that this drug may also regulate the immune system, although this approach requires further assessment. Therefore, given the association with improved nutritional status under semaglutide treatment, this GLP-1RA could be explored in other obesity-related conditions, including fatty liver disease, dyslipidemia, hypertension, and potentially even cancer.

## Conclusion

Obesity is a current public health issue that must be addressed to avoid and prevent several underlying pathologies like cardiovascular diseases, fatty liver disease, DM2, hypertension, and cancer. Therefore, lifestyle and pharmacological options must be evaluated to improve or prevent those obesity underlying pathologies. Between the pharmacological options, the GLP-1RA, originally approved for the treatment of DM2, have been demonstrated to be useful for body weight reduction in obese individuals. The main molecular mechanisms of GLP-1RA in obesity reduction include the increased production of insulin by the cAMP/PKA pathway in pancreatic β-cells, along with an augmented translocation of GLUT-4 in the cellular membrane for improved glucose homeostasis, and energy expenditure by lipolytic cycle stimulation. The GLP-1RA with better outcomes in clinical trials is semaglutide since this drug can reduce up to 15% of the baseline weight after a 2.4 mg subcutaneous weekly dose. Moreover, semaglutide has shown a reduction of cardiovascular risk factors and inflammatory proteins observed in obese individuals. Therefore, semaglutide could improve other obesity-related pathologies like cardiovascular, hypertension, or fatty liver diseases.

## Author contributions

RT-T: Conceptualization, Writing – original draft, Writing – review & editing. VR-P: Conceptualization, Writing – original draft, Writing – review & editing. SC-U: Writing – original draft, Writing – review & editing. PG-R: Writing – original draft, Writing – review & editing, Writing – original draft. EP-C: Writing – original draft, Writing – review & editing. RZ-V: Writing – original draft, Writing – review & editing. DS-R: Conceptualization, Resources, Writing – original draft, Writing – review & editing. AZ: Conceptualization, Funding acquisition, Project administration, Resources, Supervision, Writing – original draft, Writing – review & editing.
